# Assessment of Medication Compliance and Follow-Up Clinic Attendance Among Patients With Cardiovascular Diseases in the Jazan Region

**DOI:** 10.7759/cureus.63928

**Published:** 2024-07-05

**Authors:** Ibrahim M Gosadi, Mnar H Moafa, Maryam K Magfouri, Ramah M Kuriri, Wasan M Hattan, Rahaf S Othathi, Ghadi F Alsum, Lujain B Suhaqi, Ahmed Sayed, Sarah M Salih

**Affiliations:** 1 Department of Family and Community Medicine, Jazan University, Jazan, SAU; 2 Department of Medicine, Jazan University, Jazan, SAU; 3 Department of Internal Medicine/Cardiology, Faculty of Medicine, Jazan University, Jazan, SAU

**Keywords:** jazan region, saudi arabia, cvd patients, compliance, appointments, adherence, medications, cardiovascular diseases

## Abstract

Background and aim: Cardiovascular diseases are common causes of mortality in Saudi Arabia and the world. This study aims to assess medication compliance and regularity of follow-up for cardiovascular patients in the Jazan region.

Methodology: An analytical cross-sectional approach was used to target all registered cardiovascular patients attending the cardio clinic in a Jazan region hospital. Data were collected using an interview questionnaire developed by the researchers with the help of experts. The questionnaire included the patients’ sociodemographic data, clinical characteristics, disease-related data, drugs, and appointments.

Results: The study included 259 patients diagnosed with cardiac disease. About 53.7% of the patients were males. All the cases had the disease for one year or more. About 56% of the patients had no difficulty remembering their medications, while 44% had problems remembering to take them. More than half of the patients had good medication adherence, and 79.6% had good appointment adherence. Only 20.4% of patients had a poor adherence rate.

Conclusion and recommendations: The adherence rate for the patients’ medication and appointments was satisfactory due to high patient awareness. On the other hand, poor adherence was related more to non-Saudi patients.

## Introduction

The term “cardiovascular diseases” (CVDs) refers to various heart and blood vessel conditions. CVDs include heart attack, heart stroke, coronary heart disease, cerebrovascular disease, peripheral arterial disease, rheumatic heart disease, and congenital heart disease (CHD) [1]. The prevalence of coronary artery disease (CAD) among heart failure patients is 62.5% in Saudi Arabia and 60.2% in the seven Middle Eastern nations [2]. Follow-up is defined as timely monitoring of health status and guidance in a drug regimen by various methods for patients who have visited or been visited by medical staff [3]. Adherence to follow-up is typically measured as the follow-up rate, also known as the attendance rate, retesting rate, or screen rate. Multiple definitions and calculations may be used for these terms. Adherence to follow-up plays a crucial part in managing chronic diseases because it is a medical procedure characterized by long-term surveillance, together with the results of the therapy [3]. ST- and non-ST-elevation myocardial infarction (MI) patients had annual costs that were, on average, Saudi Arabian riyal (SAR) 54,877 and SAR 50,215, respectively. Each patient with unstable angina had an average yearly expense of SAR 43,838; for stable angina, it was SAR 41,640. Each ischemic stroke patient paid SAR 89,739 on average per year in expenses, including hospitalization and prescription costs (27% and 11%, respectively) [4].

CVDs are common causes of mortality in Saudi Arabia and the world [1]. This group of diseases requires many periodic appointments and medications. Therefore, it has been noticed that some CVD patients do not adhere to their appointments and medications. Non-adherence is a multifaceted problem. To get any definitive results, it is crucial to investigate various issues. The literature review on individual-related variables, healthcare provider factors, and healthcare system aspects is presented under the general heading of factors impacting medication adherence for hypertension and other diseases [5]. Some of the individual-related factors (socio-demographics, knowledge, beliefs, and factors relating to their health difficulties) and those relating to patient-physician relationships (verbal and nonverbal communication and physician communication) were found to be favorably connected to medication adherence in a meta-analysis of 106 correlational studies. In 21 experimental interventions about doctor-patient communication and treatment adherence, it was found that patients treated by physicians with poor communication skills had a 19% increased risk of treatment non-adherence [6]. Regarding factors related to the healthcare system, the general and private hospitals in Saudi Arabia are better resourced with educational and informational materials than the regional primary healthcare clinics. Patient and health education resources in primary healthcare clinics are neglected areas that need specific attention from the Saudi health system [7].

A descriptive cross-sectional study discussed difficulties relating to patient-physician relationships and views about medication. To investigate areas of worry and misunderstandings about utilizing drugs, it is vital to evaluate patients' beliefs. Interventions for education are essential to improve patient understanding and, as a result, alter their perceptions of their therapy. A crucial step in revealing the function of this relationship in patients' medication practices is to examine the nature of the doctor-patient relationship in Saudi Arabia [8].

Additionally, a novel telemonitoring enhanced care approach for chronic heart failure led to appreciable advancements in self-management in maintaining health and adhering to medication and food. The high degree of compliance with weight monitoring among study participants highlights the significance of seamless telemonitoring platforms to lower the possibility of patients losing interest in the technology [9].

In 2017, a literature review was performed using the Preferred Reporting Items for Systematic Reviews and Meta-Analyses (PRISMA) guidelines. Improved self-management has been found to boost compliance, enhance patient quality of life, advance clinical results, minimize hospital readmissions, and lower hospitalization costs [10]. A cohort study was conducted to determine the association between medication adherence levels and long-term major adverse cardiovascular events in these patients. For the post-MI population, full adherence to recommended medicines was linked to a lower rate of serious adverse cardiovascular events and cost savings, with a threshold effect at >80% adherence; to continue reaping the benefits, at least a 40% level of long-term adherence must be maintained. Fewer cardiovascular events may result from novel strategies to enhance adherence [11].

According to a multi-center prospective cohort research study, medication non-adherence after MI is frequent and starts soon after hospital discharge. People who stop taking medications that have been effective have a higher mortality risk. These findings point to the need to transfer care from the hospital to the outpatient setting to ensure that patients continue to take medications that positively impact mortality [12]. A quantitative meta-analysis of randomized trials was conducted about the effect of interventions to improve patient appointment adherence and concluded that the average rate of compliance with appointments was 58%. Also, mailed reminders, telephone calls, orientation statements, contracting with patients, and prompts from physicians were consistently helpful in reducing missed appointments [13].

Several variables have been associated with low compliance with cardiovascular therapy, including patient, physician, patient-physician relationship, treatment, environment, and illness characteristics. As a result, several therapies have been attempted to counteract these characteristics and enhance patient adherence to suggested treatments. While the findings have been mixed, there appears to be agreement that multiple-approach interventions are more successful than single-strategy ones at boosting compliance, particularly with long-term therapies. Unfortunately, no evidence suggests that all of these complicated treatments are necessary or which ones, if any, are the most beneficial [14].

Based on our literature search, no studies have been conducted to assess medication compliance and follow-up clinic attendance among patients with CVDs in the Jazan region. Our study aims to fill this gap and help evaluate the compliance with medication and regularity of follow-ups by cardiovascular patients in the Jazan region [14].

## Materials and methods

Study design and settings

This study has a cross-sectional analytical study design. It was conducted in Prince Mohammed Bin Nasser Governmental Hospital’s cardiology clinic in Jazan, Saudi Arabia between December 2022 and April 2023. The study targeted patients diagnosed with a cardiac disease. All patients diagnosed with CVD, whether males or females, above 18 years, and who were able to verbally communicate with data collectors were included. Patients who were not diagnosed with any cardiovascular condition, cardiovascular patients under the age of 18 years, patients unable to verbally communicate with data collectors, newly diagnosed cardiovascular patients who have not yet received any cardiac medications, and those who did not complete one year of follow-up were excluded. Systematic random sampling was utilized to recruit the study sample. A list of patients attending the follow-up clinics was used to generate the selection list. Every third patient was approached and recruited after securing their informed consent.

Data collection tool

The questionnaire was developed based on four major components. Firstly, demographic data of the cardiac patients were assessed, including data about age, gender, nationality, education level, marital status, accommodation type, and monthly income. Secondly, clinical data of the patients included history of comorbidity, duration since diagnosis with a cardiac condition, type of cardiac disease, cardiac symptoms, and history of congenital heart defects. The third component assessed the medication practice of the patients and inquired about the number of medications the patient takes daily, the number of doses, the number of days in which the patient did not take medications, and the patients’ compliance with taking their medication. The medication compliance was assessed using a previously developed questionnaire where the content, validity, and reliability of the questionnaire measuring medication compliance are described elsewhere [15]. The final component was related to the extent to which cardiovascular patients adhered to their appointments and included the number of appointments the patient had during the year, the number of missed appointments, the number of appointments attended, and the reasons for missing appointments. The questionnaire items were open-ended or in a categorical format. Notes were taken via interviews, and transcripts of interviews were produced to facilitate data synthesis.

Data collection process

**Figure 1:**
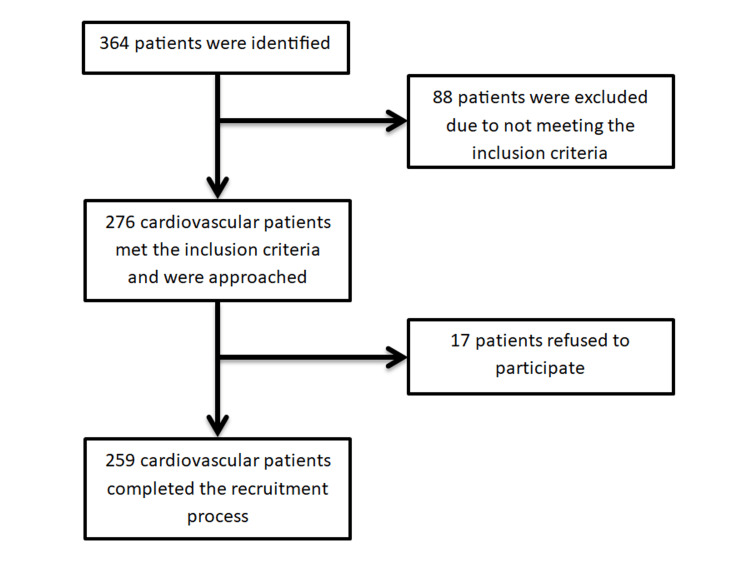
Data collection process flow chart.

After receiving administrative approval to conduct the study in the chosen Prince Mohammed Bin Nasser Governmental Hospital clinic, communications with the clinic's health workers were established to facilitate data collection. The steps to gather data from study participants are displayed in Figure 1 and are described below.

Patients attending follow-up appointments at selected clinics were identified via healthcare workers at the facility. After identifying study subjects and assessing inclusion criteria, subjects meeting them were approached, and the study principles were explained. Those interested in participating were asked to provide written consent prior to participation and were recruited in the study. Recruitment included filling out questionnaires via interviews where investigators collected data. The interviews were conducted by trained medical students.

The sample size estimation of the current investigation was based on a study conducted by Altuwairqi, which measured medication adherence among cardiac patients at a tertiary healthcare center in Riyadh, Saudi Arabia [4]. Altuwairqi concluded that 33.7% of patients had low medication adherence. The StatCal function of Epi Info (Centers for Disease Control and Prevention, Atlanta, GA) was applied to estimate the required sample size of our investigation. Assuming an expected frequency of 33.7%, a 95% confidence level, and a 5% acceptable margin of error, a sample of 343 patients was estimated to achieve the research objectives of the current investigation.

Data analysis

SPSS version 25 (IBM Corp., Armonk, NY) was used for data analysis. Descriptive analysis was utilized to summarize the main demographic and clinical variables, including frequencies and proportions for binary and categorical data, and means and standard deviation (SD) for continuous data. Medication adherence was assessed via the established scoring system of the General Medication Adherence Scale [15]. The scale is composed of 11 items and each item is given a score varying between 0 and 3. The scores of all items are summed to provide an overall adherence level, where the highest score is 33, indicating the highest adherence level, and the lowest possible score is 0, indicating a very poor adherence level. To allow a comparison of the study characteristics according to the adherence level, the median adherence score was estimated and revealed a value of 31 in the current sample. Using the median as a cut-off point, the patients were categorized into patients with lower medication adherence if they scored a value of 30 or less and were categorized as patients with higher medication adherence if they scored values varying between 31 and 33. Furthermore, to assess variation of the patients' characteristics utilizing attendance of cardiac clinic follow-ups, the patients were grouped into patients who did not miss any appointment and those who missed a minimum of one appointment. The chi-square test was used to assess the difference in medication compliance and follow-up regularity according to the sample characteristics. A p-value of 0.05 or less was presumed as the statistically significant value for the applied test.

Ethical considerations

The study was initiated after securing the Jazan Health Ethics Committee's approval (number: 22132; dated: 06/12/2022). After the identification of the patients and upon the approaching phase, an information sheet was shared with participants explaining the purpose of the study, and the participants had the right to refuse to participate or to withdraw at any stage of the study. Data collection was initiated after securing the informed consent of the patients. The participation was anonymous and the study did not involve the collection of identification data.

## Results

The total number of patients identified in the current investigation was 364. However, as displayed in Figure 1, 88 patients were excluded due to not meeting the inclusion criteria and 17 patients refused to complete the recruitment process. This led to the recruitment of a total of 259 adult subjects in the current investigation. The demographic characteristics of the participants are displayed in Table 1. The mean age of the patients was 60 (SD: 15.6) years. The proportion of male participants was higher (53.7%) than females (46.3%), and the majority were Saudis (89.2%). Most of the participants were married (79.5%), and more than half (68%) belonged to the rural areas of Jazan, with 45.9% of them living in villas. Additionally, the majority of the participants were illiterate (44%) and had a monthly income of less than 5,000 SAR. Most of the sample were housewives (34.7%). Most of them reported never having smoked (69.5%) or chewed khat (65.3%). Finally, the majority reported no health insurance (91.5%).

**Table 1:** Demographic characteristics of 259 adult subjects from Jazan, Saudi Arabia.

Variable	Frequency (proportion)
Gender	
Male	139 (53.7%)
Female	120 (46.3%)
Nationality	
Saudi	231 (89.2%)
Non-Saudi	28 (10.8%)
Marital status	
Single	16 (6.2%)
Married	206 (79.5%)
Widowed	28 (10.8%)
Divorced	9 (3.5%)
Residence	
Rural	176 (68%)
Urban	83 (32%)
Type of accommodation	
Apartment	67 (25.9%)
Villa	119 (45.9%)
Rent	29 (11.2%)
Other	44 (17%)
Education level	
Illiterate	114 (44%)
Elementary	54 (20.8%)
Intermediate	23 (8.9%)
High	29 (11.2%)
Bachelor	37 (14.3%)
Master	2 (0.8%)
Income level	
Less than 5000	181 (69.9%)
Between 5000 and 10000	38 (14.7%)
More than 10000	40 (15.4%)
Current employment status	
Governmental employee	27 (10.4%)
Private sector owned	4 (1.5%)
Private sector employee	6 (2.3%)
Military	5 (1.9%)
Student	2 (0.8%)
Unemployment	61 (23.6%)
Retired	64 (24.7%)
Housewife	90 (34.7%)
Smoking	
Current smoker	29 (11.2%)
Ex-smoker	50 (19.3%)
Never	180 (69.5%)
Khat chewing	
Current	43 (16.6%)
Ex-chewer	47 (18.1%)
Never	169 (65.3%)
Health insurance	
No	273 (91.5%)
Yes	22 (8.5%)

Cardiovascular data and health characteristics of recruited patients are displayed in Table 2. A total of 153 (59.1%) patients were diagnosed with atherosclerosis. There were 39 (15.1%) patients diagnosed with cardiomyopathy, 20 (7.7%) patients diagnosed with stroke, 16 (6.2%) patients with arrhythmia, and 11 (4.2%) with heart enlargement. Additionally, four (1.5%) patients had CHD and three (1.2%) had angina pectoris-myocardial infarctions. On the other hand, a lower percentage of patients were reported with the following conditions: two (0.8%) patients each with tumors, pulmonary edema, and rheumatic fever, and one (0.4%) patient with heart failure. Lastly, of the 258 participants, only 15 (5.8%) were unaware of their diagnosis. Regarding the patients’ cardiac symptoms, 113 (43%) participants said they had no symptoms, 69 (26.6%) reported shortness of breath, 48 (18.5%) had chest pain, 15 (5.8%) had fatigue, nine (3.5%) had palpitations, and five (1.9%) had edemas. There were 16 (6.2%) participants with congenital heart defects. For the participants who had chronic diseases, the most frequently diagnosed chronic conditions were hypertension, with 163 (62.9%) patients; diabetes, with 122 (47.1%) patients; hypercholesterolemia, with 77 (29.7%) patients; morbid obesity, with seven (2.7%) patients; and gastrointestinal and respiratory diseases, with six (2.3%) patients.

**Table 2:** Cardiovascular data and health characteristics of recruited patients.

Variables	Frequency (proportion)
Atherosclerosis	
Patient unaware of the diagnosis	15 (5.8%)
No	91 (35.1%)
Yes	153 (59.1%)
Stroke	
Patient unaware of the diagnosis	15 (5.8%)
No	224 (86.5%)
Yes	20 (7.7%)
Cardiomyopathy	
Patient unaware of the diagnosis	15 (5.8%)
No	205 (79.2%)
Yes	39 (15.1%)
Enlargement	
Patient unaware of the diagnosis	15 (5.8%)
No	233 (90%)
Yes	11 (4.2%)
Arrhythmia	
Patient unaware of the diagnosis	15 (5.8%)
No	228 (88%)
Yes	16 (6.2%)
Regurgitation	
Patient unaware of the diagnosis	15 (5.8%)
No	238 (91.9%)
Yes	6 (2.3%)
Congenital	
Patient unaware of the diagnosis	15 (5.8%)
No	240 (92.7%)
Yes	4 (1.5%)
Tumor	
Patient unaware of the diagnosis	15 (5.8%)
No	242 (93.4%)
Yes	2 (0.8%)
Heart failure	
Patient unaware of the diagnosis	15 (5.8%)
No	243 (93.8%)
Yes	1 (0.4%)
Angina pectoris-myocardial infraction	
Patient unaware of the diagnosis	15 (5.8%)
No	241 (93.1%)
Yes	3 (1.2%)
Pulmonary edema	
Patient unaware of the diagnosis	15 (5.8%)
No	242 (93.4%)
Yes	2 (0.8%)
Rheumatic fever	
Patient unaware of the diagnosis	15 (5.8%)
No	242 (93.4%)
Yes	2 (0.8%)
Do you suffer from any cardiac symptoms right now?	
No	113 (43.6%)
Shortness of breath	69 (26.6%)
Chest pain	48 (18.5%)
Edema	5 (1.9%)
Palpitation	9 (3.5%)
Fatigue	15 (5.8%)
Do you have any congenital heart defect?	
No	243 (93.8%)
Yes	16 (6.2%)
Diabetes	
No	137 (52.9%)
Yes	122 (47.1%)
Hypertension	
No	96 (37.1%)
Yes	163 (62.9%)
Hypercholesterolemia	
No	182 (70.3%)
Yes	77 (29.7%)
Morbid obesity	
No	252 (97.3%)
Yes	7 (2.7%)
Kidney diseases	
No	255 (98.5%)
Yes	4 (1.5%)
Gastrointestinal diseases	
No	253 (97.7%)
Yes	6 (2.3%)
Endocrine diseases	
No	254 (98.1%)
Yes	5 (1.9%)
Liver diseases	
No	257 (99.2%)
Yes	2 (0.8%)
Respiratory diseases	
No	253 (97.7%)
Yes	6 (2.3%)
Anemia	
No	255 (98.5%)
Yes	4 (1.5%)
Prostate diseases	
No	256 (98.8%)
Yes	3 (1.2%)
How many medicines do you take daily? How many doses? It includes medication for all the diagnosed diseases of the patient.	
0-5	99 (38.2%)
6-10	125 (48.2%)
11-15	29 (11.1%)
16-20	5 (2%)
24	1 (0.4%)
Have you been admitted to the emergency department in the last 12 months?	
No	98 (37.8%)
Yes	160 (61.8%)

Regarding the number of medications taken by the participants and their doses, 125 (48.2%) participants took six to 10 medications, 99 (38.2%) participants took zero to five medications, 29 (11.1%) participants took 11-15 medications, five (2%) participants took 16-20 medications, and one (0.4%) participant took 24 medications. Of the participants, 160 (61.8%) were admitted to the emergency department in the last 12 months, while 98 (37.8%) were not.

Factors associated with medication compliance among the recruited patients are displayed in Table 3. More than half of the sample, comprising 145 patients (56%), reported having no trouble remembering to take their medications, while 56 patients (21.9%) reported occasionally forgetting, and 30 patients (11.6%) reported forgetting their medications regularly. Additionally, 151 patients (59%) in the sample had busy daily schedules that never impaired their medication compliance, while 26 patients (10.2%) consistently forgot to take their medications. When feeling better, 31 patients (12.1%) always stopped taking their medicines, whereas 188 people (73.4%) did not. Moreover, 31 patients (12.1%) and 21 patients (8.2%) occasionally stopped taking their prescriptions because of the side effects, but more than half of the sample did so. From the sample, 192 patients (75%) continued taking their medication even after experiencing adverse side effects. In contrast to 159 patients (62.1%) who did not have trouble remembering prescriptions as they got more complex, 27 patients (10.5%) found it challenging to recall medications as they became more complex. Compared to 207 patients (81.5%) who never adjusted the dose or the frequency of their medication on their own, nine patients (3.5%) always did so. Finally, six patients (2.3%) had difficulty purchasing medication, which is a relatively modest percentage.

**Table 3:** Factors associated with medication compliance among patients with cardiovascular diseases.

Variable	Frequency (proportion)
Do you have difficulties remembering to take your medication?	
Always	30 (11.6%)
Often	25 (9.7%)
Sometimes	56 (21.6%)
Never	145 (56%)
Do you forget to take your medication due to your busy daily schedule with work, travel, meetings, family events, parties, or religious celebrations?	
Always	26 (10.2%)
Often	34 (13.3%)
Sometimes	45 (17.6%)
Never	151 (59%)
Do you discontinue your medication when you feel well?	
Always	31 (12.1%)
Often	14 (5.5%)
Sometimes	23 (9%)
Never	188 (73.4%)
Do you stop taking medication when you experience side effects such as indigestion?	
Always	21 (8.2%)
Often	12 (4.7%)
Sometimes	31 (12.1%)
Never	192 (75%)
Do you stop taking medication without prior intimation to the doctor?	
Always	19 (7.4%)
Often	11 (4.3%)
Sometimes	23 (8.9%)
Never	204 (79.4)
Do you discontinue your medication due to medication for another disease?	
Always	10 (3.9%)
Often	9 (3.5%)
Sometimes	20 (7.8%)
Never	218 (84.8)
Have you found it difficult to remember your medication when it gets more complicated?	
Always	27 (10.5%)
Often	21 (8.2%)
Sometimes	49 (19.1%)
Never	159 (62.1%)
During the past month, have you missed medicines due to disease progression and/or the addition of new medications?	
Always	14 (5.4%)
Often	12 (4.7%)
Sometimes	36 (14%)
Never	195 (75.3%)
Do you change the dose of your medication and its frequency on your own?	
Always	9 (3.5%)
Often	6 (2.4%)
Sometimes	32 (12.6%)
Never	207 (81.5%)
Have you stopped taking these medications because it is not worth the money you spent on them?	
From 1 day to 1 month	4 (1.6%)
More than 1 month to 6 months	2 (0.8%)
More than 6 months to less than a year	14 (5.5%)
A year or more	235 (92.2%)
Have you found any difficulties in buying your medication because they are expensive?	
Always	6 (2.3%)
Often	9 (3.5%)
Sometimes	17 (6.6%)
Never	225 (87.5%)

Table 4 summarizes factors affecting the patient’s adherence to attending the appointments and taking the medication. Of the participants, 96 (37.8%) had their last appointments more than one month to six months ago and 91 (35.8%) between one day to one month ago; 40 (15.7%) participants had theirs a year or more ago, 16 (6.3%) had theirs more than six months to less than a year ago, and 11 (4.3%) did not remember when their last appointment was. Since their last appointment, 193 (77.5%) patients did not stop taking their medications for even a single day, 30 (12%) patients stopped theirs for between one day and one week, and 26 (10.4%) stopped for more than a week. Regarding the reasons for patients’ non-compliance, 33 (12.8%) patients forgot, nine (3.5%) were busy, nine (3.5%) patients’ medication expired, 11 (4.3%) were tired, seven (2.7%) felt better, three (1.2%) had to travel, three (1.2%) left due to lack of information, five (1.9%) did not attend their appointment intentionally, two (0.8%) had too much medication, and two (0.8%) were hospitalized.

**Table 4:** Factors affecting the patient’s adherence to attending the appointments and taking the medication.

Variable	Frequency (proportion)
When was your last appointment in the clinic?	
0	11 (4.3%)
From 1 day to 1 month ago	91 (35.8%)
More than 1 month to 6 months ago	96 (37.8%)
More than 6 months to less than a year ago	16 (6.3%)
A year or more ago	40 (15.7%)
Since your last appointment, how many days did you not take your medication?	
0	193 (77.5%)
From a day to a week	30 (12%)
More than a week	26 (10.4%)
If there were days when you did not take your medication, please mention the reasons.	
None	172 (66.9%)
Forgetfulness	33 (12.8%)
Fatigue	11 (4.3%)
Busy	9 (3.5%)
Did not attend intentionally.	5 (1.5%)
Hospitalized	2 (0.8%)
Feeling better	7 (2.7%)
Medication expired	9 (3.5%)
Too much medication	2 (0.8%)
Lack of information	3 (1.2%)
Travel	3 (1.2%)
Lack of supervision	1 (0.4%)
Do you use reminders for your medications?	
No	219 (84.6%)
Yes	40 (15.4%)
Was there a person reminding you to take your medications?	
No	115 (44.4%)
Yes	144 (55.6%)
In case you take any extra doses or more medication than prescribed, mention the reasons.	
None	235 (90.7%)
Fatigue	11 (4.2%)
Forgetfulness	9 (3.5%)
Did not take it on purpose	1 (0.4%)
By mistake	2 (0.8%)
Nervousness	1 (0.4%)
During the last 12 months, what was the total number of your medical appointments, including all appointments in all specialties that require your attendance at the hospital (this includes appointments for clinics, radiology departments, laboratories, etc.)?	
Didn’t answer the question	16 (6.2%)
0-5	199 (76.9%)
6-10	27 (10.5%)
11-15	15 (5.8%)
16-20	1 (0.4%)
24	1 (0.4%)
From the appointments that you mentioned, how many did you miss? Why?	
None	184 (76.3%)
Did not attend intentionally	5 (2.1%)
Busy	3 (1.2%)
No appointment available	2 (0.8%)
Hospitalized	4 (1.7%)
Reminder message issues	4 (1.7%)
Transportation problem	11 (4.6%)
Forgetfulness	10 (4.1%)
Scheduling conflict	6 (2.5%)
Travel	5 (2.1%)
Prolonged waiting in the clinic	1 (0.4%)
Fatigue	3 (1.2%)
The appointment date was not clear	3 (1.2%)
During the last 12 months, what was the total number of medical appointments in cardiac clinics?	
0-5	242 (96.5%)
6-11	7 (2.8%)
12	2 (0.8%)
How many appointments in cardiac clinics did you miss? Why?	
None	199 (79.6%)
Did not attend intentionally	3 (1.2%)
Busy	2 (0.8%)
Hospitalized	3 (1.2%)
Reminder message issues	4 (1.6%)
Transportation problem	11 (4.4%)
Forgetfulness	8 (3.2%)
Scheduling conflict	6 (2.4%)
Travel	4 (1.6%)
Prolonged waiting in the clinic	2 (0.8%)
Fatigue	4 (1.6%)
The appointment date was not clear	4 (1.6%)
If you are receiving health care at another facility, please include its name.	
No	143 (55.2%)
Yes	116 (44.8%)
What language does the cardiologist physician speak?	
Arabic	250 (96.5%)
Irish	1 (0.4%)
English	8 (3.1%)
How satisfied are you with the pharmacological education of the cardiologist who treats you?	
Strongly satisfied	145 (56%)
Satisfied	67 (25.9%)
Neutral	28 (10.8%)
Dissatisfied	9 (3.5%)
Strongly dissatisfied	10 (3.9%)
Have you ever undergone any surgical intervention that affected your commitment to medication?	
No	188 (72.6%)
Yes	71 (27.4%)
Do you believe that there is a need for ongoing cardiac follow-up?	
No	20 (7.8%)
Yes	238 (92.2%)
If you feel better, do you stop attending your appointments?	
No	204 (78.8%)
Yes	55 (21.2%)
Did the clinic send you reminders?	
No	52 (20.4%)
Yes	203 (79.6%)
Do you have someone to remind you of your appointments?	
No	86 (33.3%)
Yes	172 (66.7%)
Have you ever had to reschedule appointments?	
No	212 (81.9%)
Yes	47 (18.1%)
Have you downloaded and used any health applications such as “Tawakkalna” and “Sehati” on your phone?	
No	51 (19.7%)
Yes	208 (80.3%)

At the same time, 219 (84.6%) participants did not use any reminders for their medications, while 40 (15.4%) patients used them; 144 (55.6%) participants had a person reminding them to take their medication, while 115 (44.4%) did not have anyone. Furthermore, 235 (90.7%) patients did not take extra doses or more medication, while 11 (4.2%) took extra doses due to being more tired, nine (3.5) because they forgot, two (0.8%) by mistake, and one (0.4%) took an extra dose on purpose or because they were nervous. During the last 12 months, the total number of medical appointments for participants was one to five appointments for 199 (76.9%), six to 10 appointments for 27 (10.5%), 11-15 appointments for 15 (5.8%), and 16-20 or more for two (0.8%) patients, while 16 (6.2%) participants did not answer the question. The number of appointments in cardiac clinics in the last 12 months was one to five for 242 (96%) patients, six to 10 for seven (2.8%) patients, and 12 appointments for two (0.8%) patients. Furthermore, 199 (79.6%) patients did not miss any of their cardiac clinic appointments. For patients who missed their appointment, the reasons were as follows: 11 (4.4%) patients had transportation issues; eight (3.2%) patients forgot; six (2.4%) patients had a scheduling conflict; six (2.4%) patients did not attend intentionally or were hospitalized; 14 (6.4%) patients were fatigued, traveling, had reminder message issues, or the appointment date was unclear; and four (1.6%) were either busy or wanted to avoid the prolonged wait in the clinic.

Regarding the language of the cardiologist physician, 250 (96.5%) participants said their cardiologist spoke Arabic, eight (3.1%) patients said theirs spoke English, and one (0.4%) patient’s cardiologist physician spoke Irish. Of the participants who were satisfied with the pharmacological education imparted by the cardiologist, 145 (56%) were strongly satisfied, 67 (25.9%) were satisfied, 28 (10.8%) were neutral, 10 (3.9%) were strongly dissatisfied, and nine (3.5%) were dissatisfied. In addition, 188 (72.6%) participants did not have any surgical intervention affecting their commitment to medication; in contrast, 71 (27.4%) patients underwent a surgical intervention that affected their medication adherence. Furthermore, 238 (92.2%) participants believed that they needed ongoing cardiac clinic follow-up, while 20 (7.8%) patients did not think so. Again, 204 (78.8%) participants did not stop attending their appointments if they felt better, while 55 (21.2%) stopped attending their appointments if they felt better. Also, 203 (79.6%) participants said that the clinic sent them reminders, while 52 (20.4%) said the clinic did not send them reminders. Also, regarding the reminders, 172 (66.7%) participants had someone to remind them about the appointments, while 86 (33.3%) did not have anyone to remind them. Furthermore, 212 (81.9%) participants did not reschedule any appointments, while 47 (18.1%) rescheduled some of the appointments. Lastly, 208 (80.3%) participants had health applications such as “Tawakkalna” and “Sehati” and used them, while 51 (19.7%) participants did not.

The assessment of factors associated with medication adherence among patients with cardiovascular diseases is presented in Table 5. Only nationality was statistically associated with medication adherence (p = 0.07). Among the 222 Saudi participants, 106 (47.7%) had low medication adherence. In contrast, a higher percentage was reported in the 27 non-Saudi participants, with 18 (66.7%) patients with low and nine (33.3%) with high medication adherence. On the other hand, regarding the association of medication adherence with age (p = 0.2), marital status (p = 0.121), educational level (p = 1.0), income status (p = 0.889), smoking (p = 0.216), khat chewing (p = 1.0), gender (p = 0.375), and health insurance (p = 0.824), there were no significant differences.

**Table 5:** Factors associated with medication adherence among patients with cardiovascular diseases.

Variable	Adherence		Total	P-value
	Lower adherence	Higher adherence		
Age				
Less than 60	60 (54.5%)	50 (45.5%)	110 (100%)	0.2
60 or more	64 (46.0%)	75 (54.0%)	139 (100%)	
Marital				
Married	93 (47.2%)	104 (52.8%)	197 (100%)	0.121
Not married	31 (59.6)	21 (40.4%)	52 (100%)	
Education				
Illiterate	55 (49.5%)	56 (50.5%)	111 (100%)	1
Elementary education or more	69 (50%)	69 (50%)	138 (100%)	
Income				
Less than 5000	89 (50.3%)	88 (49.7%)	177 (100%)	0.889
5000 or more	35 (48.6%)	37 (51.4%)	72 (100%)	
Smoking				
Ever smoker	42 (56%)	33 (44%)	75 (100%)	0.216
Never smoker	82 (47.1%)	92 (52.9%)	174 (100%)	
Khat				
Ever chewer	44 (50%)	44 (50%)	88 (100%)	1
Never chewer	80 (49.7%)	81 (50.3%)	161 (100%)	
Gender				
Male	62 (47%)	70 (53%)	132 (100%)	0.375
Female	62 (53%)	55 (47%)	117 (100%)	
Nationality				
Saudi	106 (47.7%)	116 (52.3%)	222 (100%)	0.07
Non-Saudi	18 (66.7%)	9 (33.3%)	27 (100%)	
Health insurance				
No	114 (50.2%)	113 (49.8%)	227 (100%)	0.824
Yes	10 (45.5%)	12 (54.5%)	22 (100%)	

The association analysis between attending appointments and related affecting factors according to the measured sample characteristics is displayed in Table 6. The age factor was statistically associated with appointments attended (p = 0.004). Among the 116 participants under 60 years old, only 45 (38.8%) had missed at least one appointment. While in the 60 years or older age group, of the 143 participants, only 31 (21.7%) had missed a minimum of one appointment. In contrast, a higher percentage was reported regarding the 60 or older patient group; more than half of the sample, 112 (78.3%) participants, had never missed an appointment. Regarding education status, among the 114 illiterate participants, only 27 (23.7%, p = 0.099) had missed a minimum of one appointment. In contrast, of the 145 participants who had completed elementary education or more, 49 (33.8%) had missed an appointment. Regarding nationality, a higher percentage of non-Saudi participants, 11 (39.3%) out of 28 participants, reported missing at least one appointment. On the other hand, among the 231 Saudi participants, only 65 (28.1%, p = 0.271) missed an appointment. Among the 79 (p = 0.459) participants who smoked ever, only 26 (32.0%) had missed an appointment; however, among the 180 participants who never smoked, 50 (27.8%) reported having missed a minimum of one appointment. Lastly, no significant differences were found among participants who ever chewed khat (p = 0.476), participants who had an income less than SAR 5,000 (p = 0.767), married participants (p = 0.868), male participants, and those who did not have health insurance (p = 1.0) in attending appointments.

**Table 6:** The association analysis between attending appointments and related affecting factors according to the measured sample characteristics.

Variable	Appointment		Total	P-value
	None missing	Missing a minimum of one		
Age				
Less than 60	71 (61.2%)	45 (38.8%)	116 (100%)	0.004
60 or more	112 (78.3%)	31 (21.7%)	143 (100%)	
Marital				
Married	145 (70.4%)	61 (29.6%)	206 (100%)	0.868
Not married	38 (71.7%)	15 (28.3%)	53 (100%)	
Education				
Illiterate	87 (76.3%)	27 (23.7%)	114 (100%)	0.099
Elementary education or more	96 (66.2%)	49 (33.8%)	145 (100%)	
Income				
Less than 5000	129 (71.3%)	52 (28.7%)	181 (100%)	0.767
5000 or more	54 (69.2%)	24 (30.8%)	78 (100%)	
Smoking				
Ever smoker	53 (67.1%)	26 (32.9%)	79 (100%)	0.459
Never smoker	130 (72.2%)	50 (27.8%)	180 (100%)	
Khat				
Ever chewer	61 (67.8%)	29 (32.2%)	90 (100%)	0.476
Never chewer	122 (72.2%)	47 (27.8%)	169 (100%)	
Gender				
Male	98 (70.5%)	41 (29.5%)	139 (100%)	1
Female	85 (70.8%)	35 (29.2%)	120 (100%)	
Nationality				
Saudi	166 (71.9%)	65 (28.1%)	231 (100%)	0.271
Non-Saudi	17 (60.7%)	11 (39.3%)	28 (100%)	
Health insurance				
No	167 (70.5%)	70 (29.5%)	237 (100%)	1
Yes	16 (72.7%)	6 (27.3%)	22 (100%)	

## Discussion

This study aimed to assess medication compliance and follow-up clinic attendance in patients with CVDs in the Jazan region. The study revealed that most of the patients displayed satisfactory adherence to the clinical follow-ups. The main factor is that more than half of the patients have someone to remind them, or the clinic sends reminders. The explanation for more Saudi patients in the study is that we collected our samples from Prince Mohammed Bin Nasser General Hospital, which is a government hospital. In line with previous studies by Raffaa et al. (2020) that measured heart failure patients’ adherence to heart failure medications and its determinants in the Aseer region, Southern Saudi Arabia, more than half of our study participants were married. This is relevant because 55.6% of participants in our study had a person they depended upon to remind them to take their medication [16].

It is essential to highlight that there are more rural participants in our study, which may explain why transportation is the most common reason for missing some appointments. It must be indicated that the education level identified in our investigation revealed that 44% of the participants were illiterate, yet, education level was not statistically associated with the level of adherence or attendance of the appointment. Although there is no clear explanation as to why education level was not associated with medication adherence or appointment attendance in the current sample, it is possible to argue that patients had sufficient medication education or knowledge about medication intake. When we compare our study with the study by Raffaa et al. (2020), there is a similarity in the findings where the association between medication adherence was also statistically non-significant (p > 0.05) [16].

The reason that 91.5% of participants had no health insurance is that the health system in the Kingdom of Saudi Arabia aims to provide comprehensive and integrated health care to the entire population in a fair and accessible manner. Health care is provided to citizens free of charge in government medical facilities according to the strategic plan to meet the needs of the health sector.

Our study demonstrated that 153 (59.1%) patients had been diagnosed with atherosclerosis, and a further study indicated that 5.5% of the population in Saudi Arabia has CAD [2]. We observed in this study that 113 (43.6%) participants had no symptoms. We suspect this is due to the high rate of patients' medication and appointment adherence. Notably, 79.6% of the participants optimally adhered to the appointments. In our study of 259 participants, only 16 (6.2%) had a congenital heart defect, compared to the results from another study that found the prevalence of CHD to be 21 per 10,000 persons, representing 34.4% of all cardiac problems diagnosed [17]. Our results demonstrated that more than half of our sample was diagnosed with hypertension; we assume that this could be due to the large number of patients who took part in our study being 60 years of age and older. As illustrated in another study, the elderly are disproportionately affected by hypertension, which is a proven risk factor for CVD [18]. Many of the participants in our study, numbering approximately 125 (48.2%), were taking six to 10 medications and, despite that, were committed to taking the medications; we presume that this might be due to the information provided by the cardiologist to patients about the risk of non-compliance with taking medications.

This study reported the prevalence of medication adherence among 259 CVD patients. Our study concluded that the number of patients committed to taking medications without difficulty remembering them is 145 (56%), compared to 30 (11.6%) patients who are not committed. In a prior study conducted in the Kingdom of Saudi Arabia, 282 patients were included. It was observed that 61% of adherent patients reported no trouble remembering to take their medications, compared to 27.7% of non-adherent patients [15]. These findings go beyond earlier studies because they demonstrate that 26% of patients in a descriptive cross-sectional study with a sample size of 200 failed to take their prescriptions as prescribed; this number was less than what Saudi Arabian researchers had discovered in earlier studies [19]. According to a previous study also conducted in the Kingdom of Saudi Arabia, 34.4% of patients had trouble remembering to take their prescriptions. In comparison, 49% of patients forgot to take their medication at least once. Overall, only 7.3% of the patients had a high medication adherence rate, with more than half of the patients having poor medication adherence [16]. The number of participants in our study who took their medications as prescribed was higher than half of the sample; this is very significant for many reasons, including increasing awareness about the importance of medication adherence, setting up an alert schedule, and assigning one of the participants' companions to issue a reminder. Compared to the number of patients who did not take their cardiovascular medications, the causes were categorized as environmental, provider, patient, and health system [20].

This study highlights the factors affecting CVD patients' adherence rate to their medications and appointments; 77.5% of participants were committed to their medication since their last appointments, probably because half of them had an accompanying person to remind them. Therefore, 90% were committed to the doses of prescribed medications, while 10% of them took extra doses or more medication if they felt more pain. However, in one year, 79% of the patients did not miss any appointments despite having up to five appointments in the cardiac clinic; this is largely due to their belief about the need for continuous follow-up and the presence of one of their family members who reminded them.

The study explored a significant difference regarding patients' nationality and medication adherence (p = 0.07). This indicates that Saudi patients have higher medication compliance than non-Saudi patients with CVDs; the reason for this could be that most non-Saudi patients pay for their medications, so they may face challenges in bearing the costs of medications. Furthermore, the study reported no correlation between medication adherence and age. Similarly, another study in Saudi Arabia reported no significant correlation between age and medication adherence (p = 0.571) [16]. In contrast, previous studies in Saudi Arabia registered a positive correlation between patients above the age of 60 and high medication adherence (p = 0.006) [4,21]. The reasons for the nonsignificant difference in our study could be that the patients over the age of 60 may be forgetful, taking many medications for other comorbidities besides CVD, such as diabetes and hypertension, and might have transportation issues. On the other hand, in patients under 60 years, the reasons for non-compliance may be jobs and a busy lifestyle. Finally, regarding medication adherence of CVD patients, income status caused no significant difference; the likely reason for this could be that the Kingdom of Saudi Arabia provides free medications through the Ministry of Health and government medical facilities for all Saudi citizens and expatriate workers in the public sector [22].

Regarding the 259 cardiovascular patients’ adherence to their appointments, 76% did not miss any appointments in cardiac clinics. Nonetheless, the remaining 24% reported suboptimal adherence to their appointment attendance where they missed a minimum of one appointment due to varying factors. Another study reported that a large number of the sample, i.e., 238 patients (92.2%), believed in the importance of ongoing cardiac follow-up, demonstrating their high awareness, which may explain their better adherence to appointments. It is critical to highlight that 204 patients (78.8%) kept attending their appointments despite improvement in their symptoms, compared to the 55 patients (21.2%) who stopped attending any further appointments if they felt better. However, the results show that 203 patients (79.6%) received a reminder for their appointments from the clinic, which may have a role in their optimal adherence. Furthermore, our results demonstrated that more than half of the sample (66.7%) had someone to remind them of the appointments, which may positively influence their better adherence. The implications of these findings are discussed in Table 4.

The association analysis between appointment attendance and related affecting factors according to the measured sample characteristics is illustrated in Table 6. A further novel finding is that only the age factor was statistically associated with appointments attended (p = 0.004). This study found higher adherence among participants who were 60 years or older; nearly half of the sample, 112 (78,3%), never missed any appointments. In contrast, of the 116 participants under 60 years old, only 45 (38.8%) missed at least one appointment. While in the age group of 60 or older, of the 143 participants, only 31 (21.7%) missed a minimum of one appointment.

The higher adherence rate among this group might be explained by the civil retirement system in Saudi Arabia initiated by the Bureau of Experts at the Council of Ministers, which stipulates that employment in government jobs ends when the employee reaches 60 years of age [23,24]. In other words, patients who were not working and, hence, not busy with life attended their appointments regularly. Finally, 17 (60.7%) non-Saudi patients out of 28 had a good adherence rate. Therefore, to what degree foreigners' health insurance influences their adherence rates remains unclear. On the other hand, among the 231 Saudi participants, only 65 (28.1%, p = 0.271) missed an appointment. Meanwhile, the remaining 166 patients (71.9%, p = 0.271) never missed an appointment. It is important to note that health care in Saudi Arabia is free for all Saudi citizens and expatriates working in the public sector, primarily through the Ministry of Health and augmented by other governmental health facilities [22].

Study strength

Because we only recruited participants at one location, only one cardiac center in the Jazan region has been included. The study results may be generalizable to similar cardiac centers in Saudi Arabia. Furthermore, another strength of our study is that we approached the patients personally through interview questionnaires, leading to a high rate of accurate data and no recurrent collection from the participants. Most importantly, we used open-ended questions, ensuring the participants expressed their opinions more freely.

Study limitations

Cardiovascular patients were questioned immediately before or shortly after attending regular check-up sessions with their private physician or in the waiting ward when most were regarded as clinically stable. Fatigue from the extended wait times may have interfered with their attention to the survey questions, and adherence to medicine and appointment rates may not have been completely accurate. The cardiac clinic was a challenging area for recruiting since both staff and heart patients were busy and pressed for time. The main limitation of this study is the number of patients excluded from our sample. A total of 89 patients had not completed a year since their diagnosis, and 27 patients could not communicate with the data collectors. At the same time, 10 patients refused to participate in the study, five had hearing challenges, and another five did not complete the questionnaire due to their appointments. Additionally, the remaining nine did not participate due to various reasons, including being patients’ companions, having Alzheimer's, simply not wanting to, being less than 18 years old, and not taking any medications. Regardless of the research study size, care must be exercised when comparing the results for various cardiovascular patient groups. Future research might examine the effects of clustering and sampling. Differences in sample size between sites may have hampered our ability to detect substantial intersite differences. Any variations in cardiac medications provided between acute inpatient and stable participants were not examined, which might have altered patients' adherence. Finally, as with many medication and appointment adherence studies, the lack of a gold standard evaluation adds ambiguity to the reported medication adherence assessments.

## Conclusions

In conclusion, this may be a promising preview of the patients’ medication and appointment adherence rates. Our study’s adherence rate was fairly satisfactory compared to other studies. We assumed that good adherence was related to factors such as patient awareness regarding improvements and ensuring that patients do not stop their medications or appointments. Also, we surmise that a high adherence rate might be due to most participants declaring that they have someone to remind them and the clinic reminder messages for appointments. Adherence was better among Saudi patients and those aged 60 years or older. More attention should be paid to accurately detecting the leading causes behind this high adherence to maintain and enhance it.

## Appendices

Questionnaire

Part One: Demographic Information

1. Gender:

- Male

- Female

2. Age:………………..

3. Nationality:

- Saudi

- Non-Saudi

4. Marital status:

- Single

- Married

- Widowed

- Divorced

5. Place of residence:

- Village

- City

6. Type of housing:

- Own apartment

- Own villa

- Renting

- Other

7. Educational level:

- Uneducated

- Elementary

- Intermediate

- Secondary

- University

- Postgraduate

8. Average income:

- Less than 5000

- Between 5000 and 10000

- More than 10000

9. Employment:

- Government employee

- Business owner

- Private sector employee

- Military

- Student

- Unemployed

- Retired

- Housewife

10. Are you a smoker?

- Yes - currently smoking

- Yes - former smoker and have quit

- Never smoked

11. Do you chew khat?

- Yes - previously and have quit

- Yes - currently

- Never

12. Do you have health insurance?

- Yes

- No

Part Two: Clinical Characteristics

13. When were you diagnosed with heart problems?

………………..

14. What cardiac disease have you been diagnosed with?

………………..

15. ‏What are the current heart-related symptoms you are experiencing?

………………..

16. Were you born with any congenital heart diseases?

- Yes

- No

17. Which of the following chronic diseases have you been diagnosed with?

- Diabetes

- Hypertension

- Dyslipidemia

- Obesity

- None

- Other:…….

18. How many medications do you take daily and how many doses? (Including all diagnosed conditions for the patient)

………………..

19. Have you been admitted to the emergency department in the last 12 months?

- Yes

- No

Part Three: Medication Adherence Information

20. Do you have any problems remembering to take your medications?

- Always

- Mostly

- Sometimes

- Never

21. Do you forget to take your medications due to your busy daily schedule, such as work, travel, meetings, family events, parties, weddings, religious celebrations., etc.?

- Always

- Mostly

- Sometimes

- Never

22. Do you stop taking your medications when you feel better?

- Always

- Mostly

- Sometimes

- Never

23. Do you stop taking medications when experiencing side effects such as indigestion?

- Always

- Mostly

- Sometimes

- Never

24. Do you stop taking medications without informing the doctor?

- Always

- Mostly

- Sometimes

- Never

25. Do you stop taking your medications because you are taking other medications for an additional condition?

- Always

- Mostly

- Sometimes

- Never

26. Do you find it difficult to remember your medications when they become more complex?

- Always

- Mostly

- Sometimes

- Never

27. In the past month, have there been instances where you forgot to take your medications due to worsening conditions or adding new medications?

- Always

- Mostly

- Sometimes

- Never

28. Do you adjust your medication dosage and frequency on your own?

- Always

- Mostly

- Sometimes

- Never

29. Have you discontinued the use of these medications because you felt they were not worth the money spent?

- Always

- Mostly

- Sometimes

- Never

30. Do you find it difficult to afford to purchase your medications?

- Always

- Mostly

- Sometimes

- Never

Part Four: Follow-Up Clinics Attendance

31. When was your last appointment in the clinic?

………………..

32. Since your last visit, how many days did you not take your medicine?

………………..

33. If there are any days when you did not take your medication, please mention the reasons that led to not taking your medications.

………………..

34. Do you use any reminder methods for taking medications?

………………..

35. Is there someone who reminds you to take your medications?

………………..

36. Did you take any extra doses or more medication than prescribed? If so, mention the reason.

………………..

Part Five: Medical Appointment Information

37. During the past 12 months, what is the total number of medical appointments you had? This includes all appointments in various specialties that require your attendance at the hospital (clinics, radiology departments, laboratories, etc.).

………………..

38. Among these mentioned appointments, how many did you miss and what were the reasons for not attending?

………………..

39. During the past 12 months, what is the total number of medical appointments in the cardiology clinics only?

………………..

40. Among these cardiology appointments, how many did you miss and what were the reasons for not attending?

………………..

41. If you receive health care at another facility, please mention its name.

………………..

42. What language does your treating cardiologist speak?

………………..

43. How satisfied are you with the medical and pharmaceutical education provided by your treating cardiologist?

- Very satisfied

- Satisfied

- Neutral

- Dissatisfied

- Very dissatisfied

44. Have you ever undergone any surgical intervention that affected your medication adherence? If yes, what was the surgical intervention and when was it?

………………..

45. Do you believe there is a need for continuous follow-up in cardiology clinics?

- Yes

- No

46. When you feel better, do you sometimes stop attending your follow-up clinics?

- Yes

- No

47. Did the clinic send you reminders about your follow-up clinics?

- Yes

- No

48. Is there someone who reminds you of your appointments?

- Yes

- No

49. Have you ever had to reschedule appointments? If yes, please mention the reason.

………………..

50. Have you downloaded any healthcare applications such as "Tawakkalna" or "Sehati" on your phone and have you used them recently?

………………..
